# A Cautionary Tale—Aortic Compression by Transesophageal Echocardiography Probe

**DOI:** 10.1016/j.jscai.2023.101128

**Published:** 2023-08-25

**Authors:** Sarah Khan, Oliver Aregullin

**Affiliations:** Congenital Heart Center, Corewell Health/Michigan State University, Helen Devos Children’s Hospital, Grand Rapids, Michigan

**Keywords:** complication, transesophageal echocardiogram, vascular compression

The use of transesophageal echocardiography (TEE) has become widespread in pediatric cardiac surgical operating rooms and cardiac catheterization labs. To limit fluoroscopy, TEE is frequently utilized for atrial septostomy and stent placement.^1^ Complications including esophageal injury, dental injury, and arrhythmia due to vasogenic response are well established in literature.^2^ A publication by Stevenson et al^3^ detailed a vascular compression incidence of 0.6% in pediatric patients, most commonly occurring intraoperatively in infants with congenital heart disease. Our report presents angiographic evidence of direct aortic compression by an appropriately sized TEE probe with resultant hemodynamic compromise.

**Figure 1:**
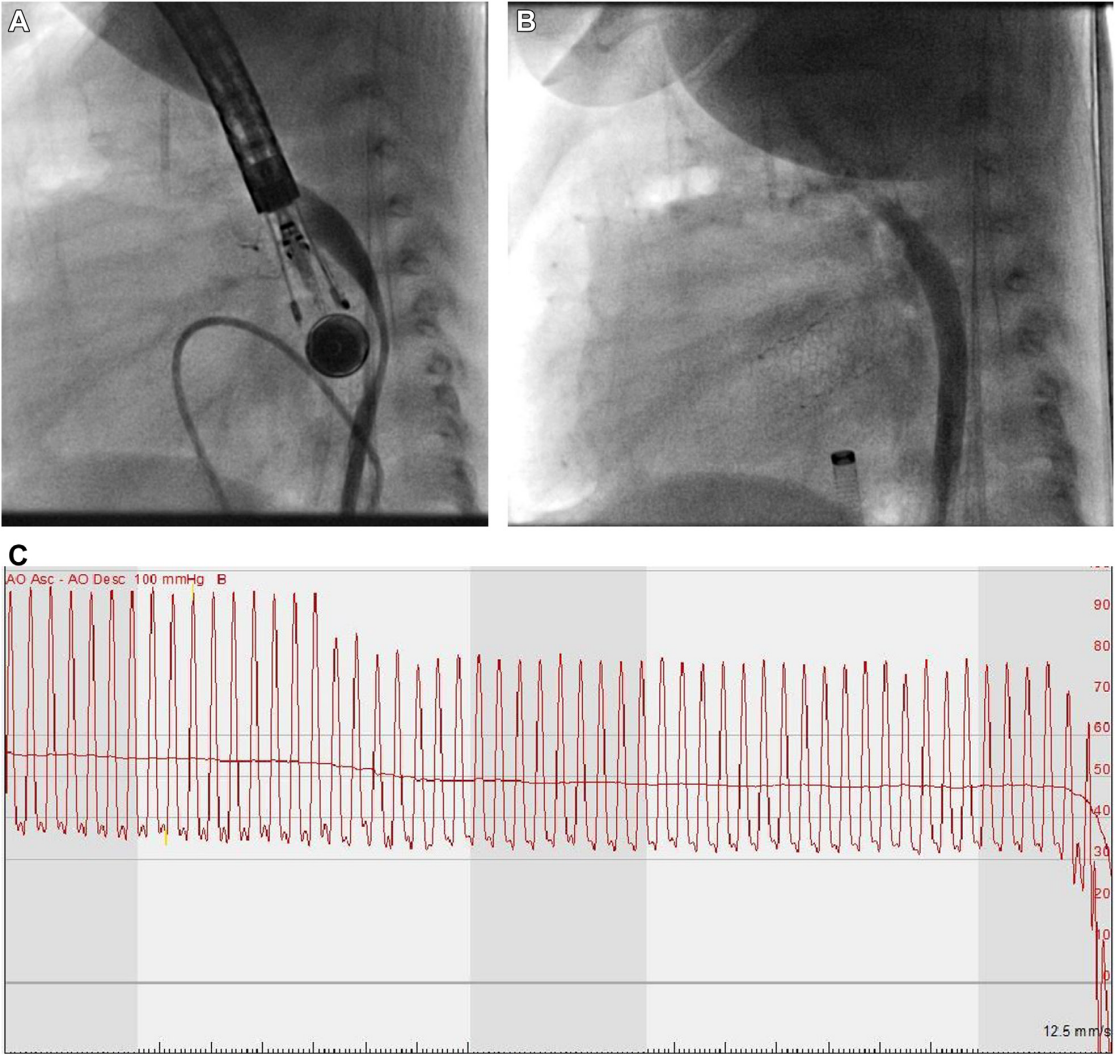
(**A**) Compression of the thoracic descending aorta by the TEE probe in anterior-posterior direction with near obliteration of aortic lumen. Probe dimensions: tip: 1.5 × 3.5 cm (0.6 × 1.4 in) W × L, shaft: 1.0 cm (0.4 in) diameter, 1 m (39.4 in). (**B**) Resolution of compression with removal of the TEE probe; contrast injection filling the widely patent thoracic descending aorta. (**C**) Femoral arterial waveform at the time of advancement of the TEE probe to mid-esophageal level with ensuing profound hypotension. TEE, transesophageal echocardiography.

Our patient was a 3-month-old, 4.2 kg, male baby with a postnatal diagnosis of critical aortic stenosis and severe mitral regurgitation, who initially underwent aortic valve balloon valvuloplasty followed by Damus-Kaye Stansel anastomosis, mitral valve repair, and Sano shunt placement as a neonate. This procedure is indicated in patients with single ventricle anatomy in which an anastomosis between the pulmonary artery and aorta provides relief of systemic outflow obstruction. The patient returned to the catheterization lab for atrial stent placement to decompress the hypertensive left atrium as well as repeat aortic balloon valvuloplasty and aortic arch angioplasty. Right heart catheterization was done through femoral venous access whereas left heart catheterization was accomplished through femoral arterial access in the usual fashion. Blood pressure was monitored through femoral arterial access. As appropriate for weight, a Philips S7-3t Pediatric TEE probe (Philips) was advanced to the mid-esophagus level. Immediately thereafter, femoral arterial tracing was lost, with ensuing profound hypotension 28/13 mm Hg. Immediate contrast injection in the descending aorta demonstrated near-complete compression of the aorta by the rotated TEE with near obliteration of the aortic lumen. With the removal of the TEE probe, repeat contrast injection demonstrated widely patent descending aorta and immediate normalization of blood pressure (Figure 1).

It is important to consider vascular compression by TEE probes as a contributing factor for decreased central perfusion and hemodynamic instability in pediatric cardiac operating rooms and catheterization labs. A case series by Lunn et al^4^ proposed anteflexion and leftward rotation of the TEE probe as the culprit. The anatomical arrangement may explain this; the esophagus lies anterior and to the left of the aorta. It then passes further to the left as it passes through the esophageal hiatus to join the stomach. In small-sized patients, patients with dilated thoracic aorta, or dilated esophagus due to gastrointestinal reflux, this close interaction can be a factor in the protrusion of the TEE probe into the aortic lumen, especially with anteflexion and rotation to the left. Intraprocedurally, it is important to monitor the probe position and ensure a neutral position when not in active use. Upper extremity arterial catheter may miss vascular compression causing downstream vascular compression. We recommend monitoring lower extremity perfusion with a pulse oximeter, blood pressure cuff, or arterial catheter.
